# Physiological and Proteomic Analyses Indicate Delayed Sowing Improves Photosynthetic Capacity in Wheat Flag Leaves Under Heat Stress

**DOI:** 10.3389/fpls.2022.848464

**Published:** 2022-03-24

**Authors:** Liwei Fei, Jinpeng Chu, Xiu Zhang, Shuxin Dong, Xinglong Dai, Mingrong He

**Affiliations:** ^1^National Key Laboratory of Crop Biology, Key Laboratory of Crop Ecophysiology and Farming System, Ministry of Agriculture and Rural Affairs, Agronomy College, Shandong Agricultural University, Taian, China; ^2^College of Resources and Environmental Sciences, Nanjing Agricultural University, Nanjing, China

**Keywords:** heat stress, photosynthetic capacity, proteomics, wheat, delayed sowing

## Abstract

**Background and Aims:**

Climate warming has become an indisputable fact, and wheat is among the most heat-sensitive cereal crops. Heat stress during grain filling threatens global wheat production and food security. Here, we analyzed the physiological and proteomic changes by delayed sowing on the photosynthetic capacity of winter wheat leaves under heat stress. Our aim is to provide a new cultivation way for the heat stress resistance in wheat.

**Methods:**

Through 2 years field experiment and an open warming simulation system, we compared the changes in wheat grain weight, yield, photosynthetic rate, and chlorophyll fluorescence parameters under heat stress at late grain–filling stage during normal sowing and delayed sowing. At the same time, based on the iTRAQ proteomics, we compared the changes of differentially expressed proteins (DEPs) during the two sowing periods under high temperature stress.

**Key Results:**

In our study, compared with normal sowing, delayed sowing resulted in a significantly higher photosynthetic rate during the grain-filling stage under heat stress, as well as significantly increased grain weight and yield at maturity. The chlorophyll a fluorescence transient (OJIP) analysis showed that delayed sowing significantly reduced the J-step and I-step. Moreover, OJIP parameters, including RC/CSm, TRo/CSm, ETo/CSm, DIo/CSm and ΦPo, ψo, ΦEo, were significantly increased; DIo/CSm and ΦDo, were significantly reduced. GO biological process and KEGG pathway enrichment analyses showed that, among DEPs, proteins involved in photosynthetic electron transport were significantly increased and among photosynthetic metabolic pathways, we have observed upregulated proteins, such as PsbH, PsbR, and PetB.

**Conclusion:**

Physiological and proteomic analyses indicate delaying the sowing date of winter wheat reduced heat dissipation by enhancing the scavenging capacity of reactive oxygen species (ROS) in flag leaves, and ensuring energy transmission along the photosynthetic electron transport chain; this increased the distribution ratio of available energy in photochemical reactions and maintained a high photosynthetic system assimilation capacity, which supported a high photosynthetic rate. Hence, delayed sowing may represent a new cultivation strategy for promoting heat stress tolerance in winter wheat.

## Introduction

The global temperature is projected to increase by 1.5–4°C by the end of this century, and climate warming has become an indisputable fact (Rasul et al., 2012; Field et al., 2014; Abbas et al., 2017; IPCC, 2018). Rising temperatures have severely affected agricultural production in many regions worldwide, including Russia (Giannakaki and Calanca, 2019), United States (Tack et al., 2015; Bergkamp et al., 2018), South Africa (Shew et al., 2020), South America (Rivelli et al., 2021), and China (Ren et al., 2011; Chen et al., 2018). Every 1°C increase from mean crop temperature causes global wheat yield losses of up to 6% (Lipec et al., 2013). Moreover, the area, frequency, and duration of heat stress during the wheat grain-filling stage has increased, threatening global wheat production and food security. Hence, enhancing the heat tolerance of winter wheat will have an important role in ensuring food security.

Photosynthesis is the process by which plants use light energy to convert absorbed carbon dioxide into organic compounds that provide energy for plant growth and development, and is an important way for green plants to accumulate dry matter (Pan et al., 2012). Heat stress affects many photosynthetic processes, including damage to photosystem II (PSII; Li et al., 2009; Mathur et al., 2014), damage to the thylakoid membrane (Prasad et al., 2008; Narayanan et al., 2015), alteration of antioxidant enzyme activity (Wang et al., 2011, 2015; Djanaguiraman et al., 2018), and reduction of chlorophyll content (Ristic et al., 2007; Prasad et al., 2008). Very high temperatures may also damage the leaf photosynthetic apparatus, leading to premature induction of leaf senescence or forced maturity at the grain-filling stage, resulting in decreased grain weight and yield (Zhao et al., 2007; Bergkamp et al., 2018; Mirosavljevi et al., 2021; Sarkar et al., 2021). At present, most studies promote crop resistance to heat stress through the application of exogenous hormones. Ethylene regulates photosynthesis through carbohydrate metabolism and antioxidant system, thus affecting the high temperature tolerance of rice (Gautam et al., 2022). Methyl jasmonate is a potential tool to protect PS II and D1 proteins in wheat plants under heat stress conditions, accelerating the recovery of photosynthetic capacity (Fatma et al., 2021). Melatonin and H_2_S protect against heat stress-induced photosynthetic inhibition by regulating carbohydrate metabolism (Iqbal et al., 2021).

In the North China Plain, global warming increased the average temperature from sowing to overwintering, and also prolongs the growth period before overwintering owing to the reduction of the period with frosts, as a result of which prompted farmers to delay the sowing time of winter wheat (Sun et al., 2007; Xiao et al., 2013, 2015; Liu et al., 2018). Delayed sowing shortens wheat growing period before overwintering, which reduced the number of main stem leaves and tillers, which reduces the number of leaves (of the main stem) and reduces the number of tillers, optimizes the nitrogen distribution in leaves (Yin et al., 2018). In addition, in short crop cycles there is less temporal dispersion between the oldest (basal) leaves and the newest (apical) ones, improves proportion of young leaves. All these changes result in higher photosynthetic rates at the individual leaf level and canopy photosynthetic nitrogen use efficiency, which resulted in the maintained grain yield (Yin et al., 2019). However, it is unclear whether delayed sowing of wheat can maintain a high photosynthetic capacity under heat stress during the grain-filling stage.

Isobaric tags for relative and absolute quantitation (iTRAQ) analysis has been used widely in proteomics quantitative research due to its unique advantages. Because proteins are the primary drivers of cellular events, proteomic analysis is becoming a powerful tool for analyzing plant reactions to environmental stresses (Budak et al., 2013) and uncovering the major processes triggered by such stresses, such as heat stress adaptation. Therefore, we conducted a comparative physiological and proteomic study of winter wheat leaves under normal and delayed sowing to reveal the mechanistic effects of delayed sowing on photosynthesis and their physiological function under heat stress, providing a theoretical basis and technical support for the cultivation of heat-tolerant winter wheat.

## Materials and Methods

### Plant Material and Treatments

The field experiment was conducted during the 2017–2018 and 2018–2019 growing seasons at the experimental station of Dongwu Village (35°97′N, 117°03′E), in Tai’an City, Shandong Province, China. The previous crop grown in the field was summer maize. The top 20 cm of the soil contained 20.61 g kg^–1^ organic matter, 1.41 g kg^–1^ total nitrogen, 29.80 mg kg^–1^ available phosphorus, and 78.89 mg kg^–1^ available potassium.

According to studies on the high-yield, high nitrogen use efficiency cultivation potential of delayed sowing in winter wheat (Dai et al., 2013; Yin et al., 2018; Zhu et al., 2019), the large ears and low tillering capacity cultivar Tainong 18 and a planting density of 405 plants m^–2^ were selected for this experiment. Wheat was sown on two dates, 8 and 22 October (normal and delayed sowing, respectively), both sowing dates were treated with heat stress from 19 days after anthesis and lasted for 3 days. There was 1 day difference in flowering time between the two sowing dates, the growth process of wheat under each treatment was shown in Supplementary Table 1. The rainfall and average temperature during the 2017–2018 and 2018–2019 growing seasons are shown in Supplementary Figure 1. The cumulative temperature above 0°C for the whole growing season, for the normal and delayed sowing dates, was 2,351.7 and 2,083.2°C d in 2017–2018 and 2,408.3 and 2,179.5°C d in 2018–2019, respectively. The cumulative temperature above 0°C prior to wintering, for the normal and late-sown treatments, was 632.8 and 401.2°C d in 2017–2018 and 623.1 and 394.4°C d in 2018–2019, respectively.

The experiment adopts a completely random arrangement design, each sowing date × thermal treatment combination repeated three times, a total of six plots, and the test area of each plot is 12 m^2^. Data from subsequent physiological experiments were analyzed based on three biological replicates in single year. The base application of pure nitrogen 120 kg.hm^–2^, P_2_O_5_ 120 kg.hm^–2^, and K_2_O 120 kg.hm^–2^ before sowing, and the topdressing of pure nitrogen 120 kg.hm^–2^ at the jointing stage. Other management measures were the same as for high-yield fields. To ensure consistency in the subsequent experiments, we selected 80 healthy and uniform plants during the flowering period of each test plot to mark them for the determination and analysis of various indicators.

### Field Warming System

**FIGURE 1:**
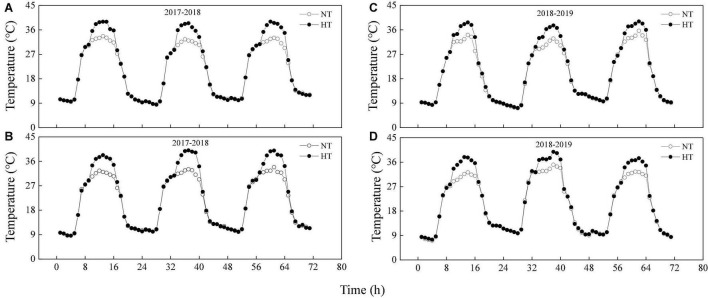
The average temperature of the air at the flag leaf layer between delayed sowing and normal sowing during heat stress (19–21 DAA). **(A,B)** Are delayed sowing and normal sowing in the 2017–2018 growing seasons, respectively. **(C,D)** Are delayed sowing and normal sowing in the 2018–2019 growing seasons, respectively. NT, Natural temperature; HT, Temperature under heat stress.

Heat stress was induced with an open field warming system using industrial high-power heaters (20 KW) to generate hot air delivered via a fan to a general air conveying belt and then to branched air belts placed between wheat rows. The hot air was blown through tubers and distributed through the field to uniformly increase the temperature. The heat treatment was carried out from 19 to 21 days after anthesis (DAA), from 10:00 to 16:00 every day. A temperature and humidity recorder (RC-4HA/C; Elitech, Puteaux, France) was fixed at the flag leaf layer, and data were recorded every 1 min. During heat stress (10:00 to 16:00), the temperature of the normal and delayed sowing were 4.75 and 5.19°C higher than the ambient temperature at the time, respectively, while the humidity of the normal and delayed sowing were maintained at about 40.07 and 40.81%, respectively (Figure 1 and Supplementary Figure 2). We found that the leaves temperature was about 1.41°C lower than the air temperature when measuring photosynthetic parameters, but there was no significant difference between normal and delayed sowing (Supplementary Figure 3). During heat stress (19–22 DAA), the average daily temperatures of the normal and delayed sowing were 22.65 and 22.95°C in 2017–2018, respectively, while the average daily temperatures of the normal and delayed sowing were 21.42 and 22.07°C in 2018–2019, respectively (Figure 1). Additionally, during the whole grain-filling period, the average daily temperatures of the normal and delayed sowing were 22.90 and 23.10°C in 2017–2018, respectively, while the average daily temperatures of the normal and delayed sowing were 20.55 and 20.64°C in 2018–2019, respectively (Supplementary Figure 1).

### Photosynthetic Rate Measurements

Healthy and uniform flag leaves of wheat plants selected to measure the net photosynthetic rate every 5 days from 15 DAA to maturity; 15 DAA represented the time before heat treatment, 20 DAA represented the time during heat treatment, and 25, 30, and 35 DAA represented the times after heat treatment. We measured 10 plants in the same area. The photosynthesis measurements were taken at 09:00–10:00 on 20 DAA, and the other measurements were taken at 09:00–11:30 when it was sunny using a CIRAS-3 portable photosynthesis measurement system (PP Systems, Amesbury, MA, United States).

### The Autioxidant Enzyme Activities and Malondialdehyde Content Measurements

In the 2018–2019, Healthy and uniform ten flag leaves of wheat plants were selected every 5 days from 15 DAA to maturity to maturity and stored at −80°C. Superoxide dismutase (SOD) activity was determined by NBT photochemical reduction method. Peroxidase (POD) activity was determined by guaiacol method. Catalase (CAT) activity was determined by hydrogen peroxide method. Malondialdehyde (MDA) content was determined by referring to Zhao et al. (1994).

### Chlorophyll Fluorescence Parameter Measurements

Chlorophyll fluorescence parameters were measured on the third day before and after heat stress. Before the measurement, the wheat flag leaf leaves were dark-adapted for 30 min, and then irradiated with saturated pulsed light (PFD = 3000 μmol m^–2^ s^–1^) for 1 s. We measured the OJIP curve and related parameters with an M-PEA continuous excitation fluorometer (Hansatech Instruments Ltd., Pentney, United Kingdom) (Strasser, 1997). We measured 10 plants in the same area. According to the method of Öz et al. (2014), the OJIP curve was standardized and analyzed using the JIP-test.

### Grain-Filling Dynamics, Grain Yield and Yield Components

We selected 300 healthy and uniform plants during the flowering period of each test plot to mark them in the 2018–2019. Thirty panicles were sampled from 5 DAA and then every 5 days until wheat matured. The grain filling process was fitted by Logistic equation, as follows:


$$y=\frac{z}{1+w{\text{e}}^{-mt}}$$

where y was grain weight, t was post-flowering time (d), and z, w, and m were fitting parameters of the equation.

In each plot, plants were harvested from an area of 2.0 m by six rows (3 m^2^) to determine the grain yield and its components. The standard moisture content was 13%. And at the same time, spike number per unit area, grain number per spike and grain weight were recorded.


$$\begin{array}{c} \mathrm{Grain}\mathrm{number}\mathrm{per}\mathrm{unit}\mathrm{area}=\mathrm{spike}\mathrm{number}\mathrm{per}\mathrm{unit}\mathrm{area}\\ \times \mathrm{grain}\mathrm{number}\mathrm{per}\mathrm{spike}. \end{array}$$

### Protein Preparation

Ten flag leaves were stored on the fourth day after heat treatment (25 DAA) in the same area. They were ground in liquid nitrogen and transferred to centrifuge tubes, and then sonicated on ice in lysis buffer (1 M sucrose, 0.5 M Tris 8.0, 0.1 M KCl, 50 mM ascorbic acid, 1% NP40, 1% sodium deoxycholate, 10 mM EDTA, 10 mM dithiothreitol, 1% protease inhibitor cocktail, and 1% phosphatase inhibitor cocktail). After centrifugation (16,000 × *g*, 4°C, 10 min), debris was removed and the protein was precipitated with cold 0.1 M ammonium acetate/methanol for 12 h at −20°C. After centrifugation (16,000 × *g*, 4°C, 10 min), the remaining precipitate was washed three times with cold acetone. The protein was redissolved in buffer (8 M urea, 50 mM Tris 8.0, 1% NP40, 1% sodium deoxycholate, 10 mM EDTA, 5 mM dithiothreitol, 1% protease inhibitor cocktail, and 1% phosphatase inhibitor cocktail) and the protein concentration was determined with a 2D Quant Kit (GE Healthcare, United States) following the manufacturer’s protocol.

### Trypsin Digestion and Isobaric Tags for Relative and Absolute Quantitation Labeling

We transferred 500 μg of protein from each sample to a new test tube. The test tubes were each adjusted to an equal volume with lysis buffer, and then incubated at 30°C for 30 min. After cooling to room temperature, 30 mM iodoacetamide was added, and the mixture was incubated in the dark at room temperature for 45 min. Then, five volumes of cold acetone were added to the mixture and incubated overnight at 20°C. After incubation, the mixture was centrifuged at 2,000 × *g* at 4°C for 10 min. The supernatant was removed, and the pellet was resuspended in cold acetone and washed three times. The precipitate was collected, and 300 μL of 0.1 M tetraethylammonium bromide (TEAB) was added to the protein after air-drying, after which ultrasonication on ice was performed to promote the dissolution of the protein precipitate. Then, 20 μg of trypsin was added and incubated overnight at 37°C. After incubation, 1% trifluoroacetic acid was added to terminate the reaction. After trypsinization, the samples were collected via centrifugation for 12 min and vacuum freeze-drying. Peptides were reconstituted in 50 μL of 0.5 M TEAB solution according to the manufacturer’s protocol for 8-plex iTRAQ reagent (Applied Biosystems, United States). The peptide mixtures were then incubated for 2 h at 25°C and then pooled, desalted, and dried using vacuum centrifugation. We used the 117 and 119 tags for the control and delayed sowing treatments, respectively.

### High-Performance Liquid Chromatography and Liquid Chromatography Tandem Mass Spectrometry

The peptide mixtures of each sample were fractionated by high-pH reverse-phase high-performance liquid chromatography using an XBridge Shield C18RP column (4.6 mm × 250 mm, 3.5 μm; Waters, United States). Then, according to the peak range displayed under an ultraviolet lamp, ∼60 components of the eluted peptide were combined into 15 components, and the vacuum concentrator was drained. Next, the dried peptide fractions were dissolved in solvent A and then analyzed by nano liquid chromatography tandem mass spectrometry (MS/MS) using an UltiMate 3000 RSLCnano system (Dionex, United States) coupled to an Orbitrap Fusion mass spectrometer (Thermo Scientific, United States). The MS scan range was set to *m/z* = 375–1800, and intact peptides were detected at a resolution of 120,000; peptides were selected for MS/MS using 30% normalized collision energy, and the MS/MS scan was set to *m/z* = 100–1800 with a resolution of 30,000. The automatic gain controls were set as 3E6 and 1E5 for MS/MS and MS, respectively. These scans targeted the 20 most abundant ions in each survey with a dynamic exclusion time of 30 s. The data-dependent acquisition procedure was applied.

### Protein Function Annotation and Data Analysis

The specific proteomic analysis method was provided by Micrometer Biotech Company (Hangzhou, China). Data were analyzed statistically using one-way analysis of variance and Duncan’s multiple range test with SPSS 25 (IBM Corp., United States), and the experimental years were analyzed separately. Differences were considered significant at *P* < 0.05.

## Results

### Changes in Grain Number, Grain Weight, and Grain Yield Under Heat Stress

There was no significant difference in grain weight of delayed and normal sowing during 0–20 DAA. But the grain weight of delayed sowing was significantly higher than that of normal sowing during 25–40 DAA (after heat stress), the fitting grain weight increased by 4.11, 11.43, 15.95, and 18.19%, respectively (Supplementary Figure 4).

**FIGURE 2:**
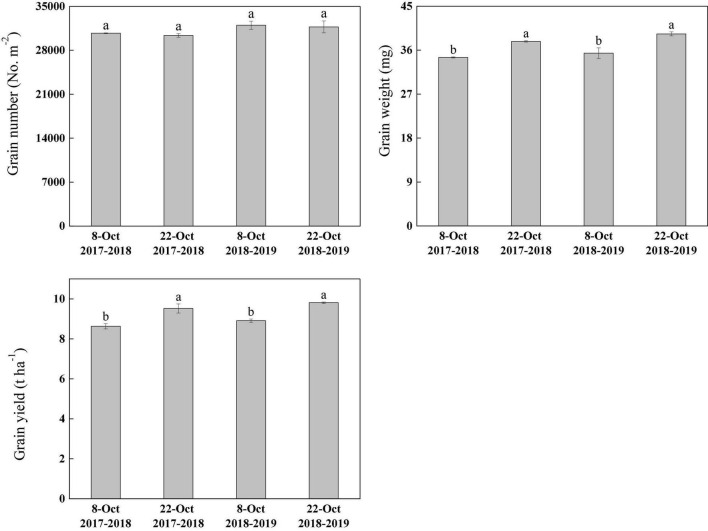
Effect of delayed sowing on grain number, grain weight, and grain yield of wheat under heat stress at late grain–filling stage. Values are means of three replicates per treatment. Vertical bars indicate standard error. Different letters denote statistical differences by LSD test (*P* < 0.05) between treatments for each parameter in the same year.

Under heat stress, sowing date significantly affected the grain weight and yield, but there was no difference in grain number per unit area. Compared with normal sowing, the grain weight increased by 9.51 and 11.16% under delayed sowing and grain yield increased by 10.31 and 10.10% in the 2017–2018 and 2018–2019 growing seasons, respectively (Figure 2).

### Changes in the Photosynthetic Rate of Wheat Flag Leaves Under Heat Stress

**FIGURE 3:**
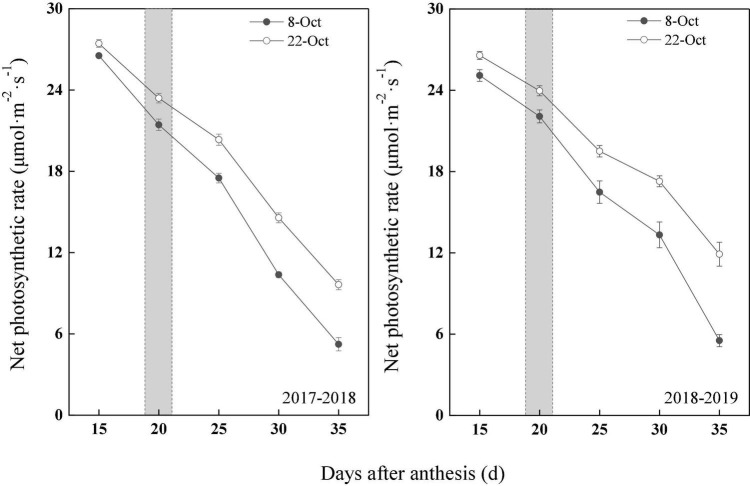
Effect of delayed sowing on photosynthetic rate of wheat flag leaves under heat stress at late grain–filling stage. With a gray area from 19 to 21 DAA. Values are means of three replicates per treatment. Vertical bars indicate standard error.

With wheat growth and development after anthesis, the photosynthetic rate showed a downward trend; however, the photosynthetic rate of the delayed sowing treatment was significantly higher than that of the normal sowing treatment (Figure 3). In 2017–2018, compared with normal sowing, the net photosynthetic rate in flag leaves after anthesis (15–35 days) increased by 3.39, 9.18, 16.19, 40.51, and 84.08% under delayed sowing. Similarly, in 2018–2019, the net photosynthetic rate in flag leaves after anthesis (15–35 days) increased by 5.90, 8.57, 18.30, 29.67, and 115.38%.

### Changes in the Autioxidant Enzyme Activities and Malondialdehyde Content of Wheat Flag Leaves Under Heat Stress

**FIGURE 4:**
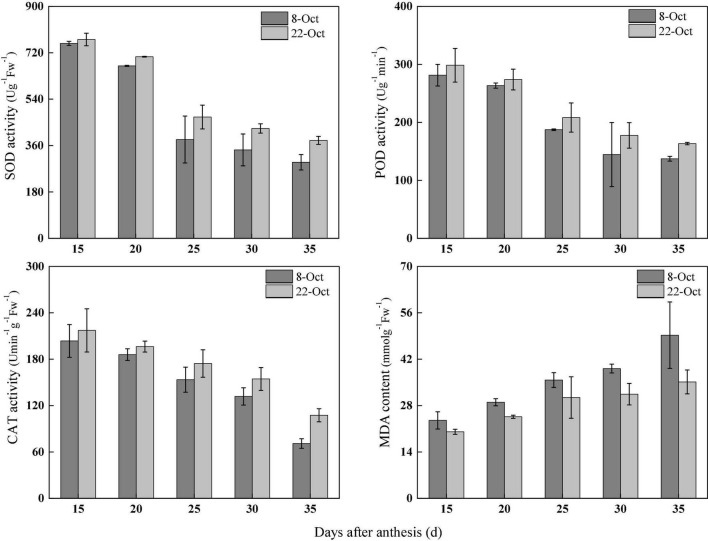
Effect of delayed sowing on antioxidant enzymes activity and MDA content of wheat flag leaves under heat stress at late grain-filling stage.

Under heat stress, in 2018–2019, compared with normal sowing, the SOD activity in flag leaves after anthesis (15–35 days) increased by 2.01, 5.33, 22.74, 24.23, and 28.72% under delayed sowing. Similarly, POD activity increased by 6.14, 3.99, 11.20, 22.85, and 19.04%, CAT activity increased by 6.72, 5.61, 13.61, 17.07, and 51.52% and the MDA content decreased by 14.65, 15.09, 14.80, 19.72, and 28.62%, respectively, under delayed sowing (Figure 4).

### Relative Variable Fluorescence Intensity of Photosystem II in Wheat Flag Leaves Under Heat Stress

**FIGURE 5:**
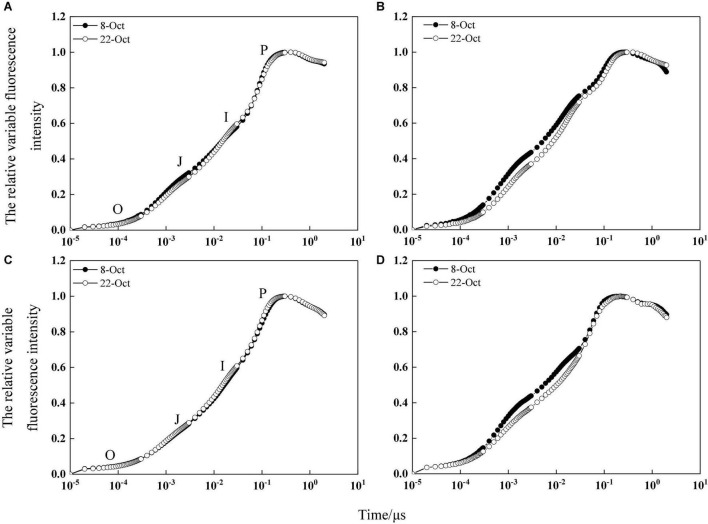
JIP-test curve of relative variable fluorescence intensity before heat stress. **(A,C)** Are JIP-test curve of relatively variable fluorescence on the third day before heat stress in the 2017–2018 and 2018–2019 growing seasons, respectively. **(B,D)** Are JIP-test curve of relatively variable fluorescence on the third day after heat stress in the 2017–2018 and 2018–2019 growing seasons, respectively.

According to the OJIP curve of flag leaves at the grain-filling stage, the JIP-test curve before heat stress was not affected by delayed sowing, indicating that sowing date had little effect on the PSII donor and acceptor sides (Figures 5A,C). However, after heat stress, the JIP-test curve of the delayed sowing treatment was significantly lower than that of the normal sowing treatment at the J-step and I-step, indicating that the primary (Q_*A*_)-to-secondary (Q_*B*_) acceptor plastoquinone electron transfer process in the leaves of the normal sowing treatment was severely inhibited under heat stress (Figures 5B,D). Moreover, sowing date significantly affected the amount of active PSII reaction centers per cross section (RC/CSm), trapped energy flux per CSm (TRo/CSm), electron transport flux per CSm (ETo/CSm), and dissipated energy flux per RC (DIo/CSm) in winter wheat flag leaves. In 2017–2018 under heat stress, RC/CSm, TRo/CSm, and ETo/CSm increased by 3.95–43.93%, 2.73–10.65%, and 3.22–38.79%, respectively, while DIo/CSm decreased by 1.76–6.87% under delayed sowing compared to normal sowing; similarly, in 2018–2019, RC/CSm, TRo/CSm, and ETo/CSm increased by 4.27–47.54%, 2.94–9.30%, and 4.23–37.39%, while DIo/CSm decreased by 2.73–7.74% (Table 1).

**TABLE 1:** Effect of delayed sowing on the energy fluxes per excited cross section of wheat flag leaves under heat stress at late grain–filling stage.

Growth season	Index	Sowing date	15 DAA	20 DAA	25 DAA	30 DAA	35 DAA
2017–2018	RC/CSm	8-October	19285.68b	17863.63b	17077.54b	13463.21b	7763.16b
		22-October	20047.33a	19897.62a	18870.17a	15878.17a	11173.17a
	TRo/CSm	8-October	16955.72b	16578.14b	14584.17b	14734.01b	13752.06b
		22-October	17418.52a	17152.95a	16137.37a	15934.18a	14809.25a
	ETo/CSm	8-October	12668.88b	12098.21b	9057.27b	7653.12b	4748.81b
		22-October	13256.17a	12487.26a	9967.48a	9150.19a	6591.07a
	DIo/CSm	8-October	4249.36a	4353.46a	4412.05a	4678.32a	5179.48a
		22-October	4138.46b	4276.73a	4302.16b	4459.13b	4823.46b
2018–2019	RC/CSm	8-October	19615.22b	18297.28b	17731.04a	14577.38b	8678.773b
		22-October	20452.28a	21906.44a	17748.46a	17174.87a	12804.46a
	TRo/CSm	8-October	17603.53b	16336.50b	15004.00b	14338.33b	12047.50b
		22-October	18121.64a	17197.57a	15987.25a	15753.25a	15054.25a
	ETo/CSm	8-October	13194.00b	12568.00b	9948.67b	8217.67b	5204.00b
		22-October	13791.27a	13099.71a	10769.75a	9801.00a	7149.75a
	DIo/CSm	8-October	4425.53a	4446.20a	4702.20a	4744.75a	4921.00a
		22-October	4304.73b	4351.00b	4549.20b	4618.00b	4796.43b

*DAA, Days of anthesis. Different values followed by different letters in the same columns are significantly different among the treatments at 0.05 level in the same year.*

Based on the absorbed light energy distribution ratio, sowing date significantly affected the maximal photochemical efficiency of PSII (ΦPo), excitation efficiency of electron transport beyond Q_*A*_ (ψo), quantum yield for electron transfer (ΦEo), and quantum ratio for heat dissipation (ΦDo) (Table 2). In 2017–2018 under heat stress, ΦPo, ψo, and ΦEo increased by 1.34–6.25%, 1.05–32.51%, and 2.61–39.41%, respectively, while ΦDo decreased by 6.44–13.37% under delayed sowing compared to normal sowing; similarly, in 2018–2019, ΦPo, ψo, and ΦEo increased by 1.65–6.14%, 1.26–30.46%, and 2.50–37.14%, while ΦDo decreased by 6.53–14.65%. Thus, under heat stress, a higher proportion of energy in the flag leaves enters electron transfer under delayed sowing, which increases the energy available in photochemical reactions, whereas a higher proportion of energy in the flag leaves is used for heat dissipation under normal sowing.

**TABLE 2:** Effect of delayed sowing on the energy distribution proportion of wheat flag leaves under heat stress at late grain–filling stage.

Growth season	Index	Sowing date	15 DAA	20 DAA	25 DAA	30 DAA	35 DAA
2017–2018	ΦPo	8-October	0.7856b	0.7410b	0.6712b	0.6342b	0.5932b
		22-October	0.7961a	0.7658a	0.7097a	0.6738a	0.6050a
	Ψo	8-October	0.7250b	0.6524b	0.5444b	0.5402b	0.3742b
		22-October	0.7326a	0.7051a	0.6339a	0.6324a	0.4958a
	ΦEo	8-October	0.5795b	0.5266b	0.4389b	0.4252b	0.2747b
		22-October	0.5946a	0.5649a	0.5113a	0.5075a	0.3829a
	ΦDo	8-October	0.1940a	0.2319a	0.2632a	0.2812a	0.3198a
		22-October	0.1815b	0.2048b	0.2293b	0.2436b	0.2827b
2018–2019	ΦPo	8-October	0.7980b	0.7580b	0.6958b	0.6856b	0.6632b
		22-October	0.8112a	0.7818a	0.7358a	0.7277a	0.6933a
	Ψo	8-October	0.7527b	0.6765b	0.5978b	0.5790b	0.4107b
		22-October	0.7622a	0.7377a	0.6853a	0.6756a	0.5358a
	ΦEo	8-October	0.6035b	0.5460b	0.4832b	0.4555b	0.3021b
		22-October	0.6186a	0.5915a	0.5513a	0.5425a	0.4143a
	ΦDo	8-October	0.2020a	0.2398a	0.2902a	0.3019a	0.3494a
		22-October	0.1888b	0.2148b	0.2477b	0.2598b	0.3067b

*DAA, Days of anthesis. Different values followed by different letters in the same columns are significantly different among the treatments at 0.05 level in the same year.*

### Proteomic Analysis of Leaves in Response to Sowing Date Under Heat Stress

To explore the impact of sowing date under heat stress at the late grain-filling stage in winter wheat, we analyzed normal and delayed sowing treatment groups (three independent biological replicates each) using iTRAQ labeling proteomics. We identified 5,896 proteins, of which 4,411 were quantified (Supplementary Figure 5A). The groups were compared according to a fold-change >1.500 (significant increase) or <0.667 (significant reduction), as well as Student’s *t*-test using *P* < 0.05 to indicate significance. In the delayed sowing group, we identified 56 upregulated proteins and 83 downregulated proteins (Supplementary Figure 5B); we subsequently screened these 139 differentially expressed proteins (DEPs; Supplementary Figures 5A,B).

According to UniProt^1^, Gene Ontology (GO)^2^, and the biological processes related to the proteins, DEPs were divided into seven categories: protein activity (36, 25.90%), metabolism (29, 20.86%), photosynthesis (20, 14.39%), stress response (15, 10.79%), cell structure (12, 8.63%), transportation (13, 9.35%), and other categories (Supplementary Figure 5C and Supplementary Table 2). Thus, sowing date impacts multiple biological processes in wheat leaves under heat stress.

According to functional classification, upregulated proteins tended to be related to photosynthesis and stress response, whereas downregulated proteins tended to be related to cell structure, protein activity, metabolism, and transportation. Among the proteins related to stress response, 1 protein related to heat stress was significantly upregulated (A0A3B6PNM7), and 5 proteins related to oxidation-reduction process were significantly upregulated, such as glutathione reductase (GR) (A0A3B6JM67). Since photosynthesis showed a significantly increased trend with physiological indicators, we focused on the analysis of DEPs related to photosynthesis (Supplementary Table 2).

### Proteins Involved in Photosynthesis

We identified 20 DEPs related to photosynthesis, of which 12 were significantly upregulated and eight were significantly downregulated (Table 3). Thus, delayed sowing may have a mitigating effect on heat stress at the late grain-filling stage. Furthermore, seven DEPs were related to the synthesis of chlorophyll precursors, of which four were significantly downregulated and three were significantly upregulated (A0A3B6LW30, A0A3B6U5D2, and A0A3B5Z1L6). Ten DEPs were related to photosynthetic electron transport, of which six were significantly upregulated (S4Z3A1, A0A3B6NU25, A0A3B6QKC9, A0A3B6EN87, A0A3B6NW49, and P60162). Finally, three DEPs were related to the Calvin cycle, all of which were significantly upregulated (A0A3B6MXQ2, A0A3B6JP0, and ZA0A2).

**TABLE 3:** Photosynthesis-related proteins differentially expressed under heat stress at late wheat grain–filling stage.

Protein accession (Photosynthesis/Respiration)	Number	Protein description	22-October/8-October ratio	22-October/8-October *P* value
**Synthesis of chlorophyll precursors**	7			
A0A3B6LW30		Mg-protoporphyrin IX chelatase	1.56	0.0035
A0A3B6U5D2		“Chlorophyll a-b binding protein, chloroplastic”	1.52	0.0017
A0A3B5Z1L6		Uncharacterized protein	1.51	0.0276
A0A3B6MQD6		Uncharacterized protein	0.63	0.0425
A0A3B6LKD1		PAP_fibrillin domain-containing protein	0.57	0.0137
A0A3B6C222		PAP_fibrillin domain-containing protein	0.46	0.0000
A0A1D5UUP8		PAP_fibrillin domain-containing protein	0.44	0.0034
**Photosynthetic electron transport**	10			
S4Z3A1		Photosystem II reaction center protein H	2.08	0.0000
A0A3B6NU25		Uncharacterized protein	1.92	0.0005
A0A3B6QKC9		PKS_ER domain-containing protein	1.71	0.0202
A0A3B6EN87		CAAD domain-containing protein	1.63	0.0130
A0A3B6NW49		Uncharacterized protein	1.53	0.0102
P60162		Cytochrome b6	1.51	0.0002
A0A3B5XW56		Uncharacterized protein	0.54	0.0021
A0A218LW52		NADH-plastoquinone oxidoreductase subunit I (Fragment)	0.56	0.0004
A0A3B6TNU5		Uncharacterized protein	0.21	0.0178
A0A3B6RNY5		Uncharacterized protein	0.28	0.0001
**The Calvin cycle**	3			
A0A3B6MXQ2		FMN hydroxy acid dehydrogenase domain-containing protein	2.04	0.0138
A0A3B6JJ08		ATPase_AAA_core domain-containing protein	1.57	0.0005
A0A2P0ZG21		Ribulose bisphosphate carboxylase large chain (Fragment)	1.53	0.0040

### Functional Enrichment Analysis of Differentially Expressed Proteins

**FIGURE 6:**
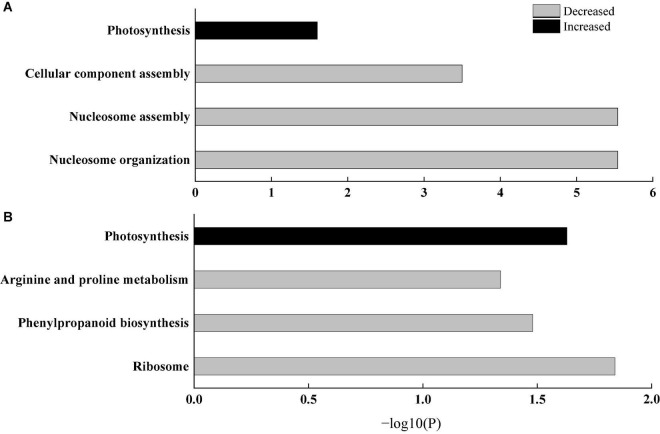
Enrichment analysis of differential protein. **(A)** GO biological process analysis. **(B)** KEGG pathway analysis. The bars represent –log10(P) where P represents the Fisher’ exact test *P*-values.

**FIGURE 7:**
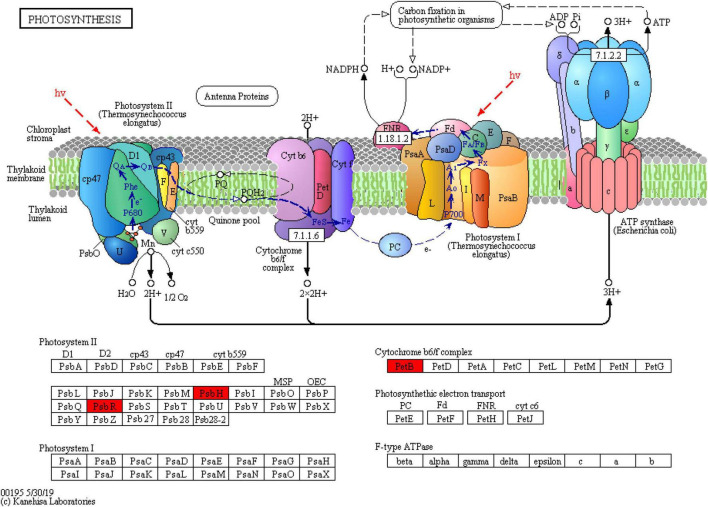
DEPs involvement photosynthetic were mapped in the metabolic pathway by KEGG analysis (red represents upregulated protein).

To further explain the response of wheat flag leaves under heat stress to the two sowing date treatments, we conducted GO biological process and KEGG pathway enrichment analyses. Among biological processes, cellular component assembly, nucleosome assembly, and other proteins related to cell structure decreased significantly, whereas proteins related to photosynthesis increased significantly, mainly those related to photosynthetic electron transport (e.g., GO: 00159796) (Figure 6A). Thus, delaying sowing of wheat can increase leaf photosynthesis and reduce cell structure activity under heat stress by increasing photosynthetic electron transport. Moreover, KEGG pathways such as ribosomal protein, phenylpropanoid biosynthesis, and arginine and proline metabolism were significantly downregulated, whereas pathways related to photosynthesis were significantly upregulated (Figure 6B). Among photosynthetic metabolic pathways, we only observed upregulated proteins (PsbH, PsbR, and PetB); among them, PsbH and PsbR are related to PSII and PetB is related to the cytochrome b6f complex (Figure 7). These results further confirm that delaying sowing of wheat is beneficial to leaf photosynthesis under heat stress.

## Discussion

As less than 50% of tillers in winter wheat produces grain-bearing ears (Whaley et al., 2000), tiller mortality wastes a large amount of wheat yielding resources (Berry et al., 2003). Delayed sowing significantly reduced the tiller number before winter and the maximum tiller number before jointing, reducing the number of leaves (main stem) and improving the tiller survival percentage (Yin et al., 2018). Meanwhile, in a shorter crop cycle, the time dispersion between the oldest (base) leaf and the newest (top) leaf was less. Therefore, under the same cultural environmental conditions, delayed sowing optimizes the nitrogen content in leaves to increase photosynthetic rate (Yin et al., 2019). This is consistent with our study, compared with normal sowing, delayed sowing resulted in a significantly higher photosynthetic rate and grain weight during the grain-filling stage under heat stress, thus ensuring a significant increase in grain weight and yield under heat stress.

Cereal crop growth after flowering depend on carbon remobilization from stems and photosynthesis from flag leaf and spike (Simpson et al., 1983; Blum, 1985; Elizabete et al., 2017; Townsend et al., 2018). Photosynthesis is sensitive to heat stress, and its performance under adverse conditions can reflect the stress tolerance of plants (Chaves et al., 2003; Lu et al., 2017; Mahrookashani et al., 2017). Therefore, it suggests that such delayed sowing wheat can maintain a higher photosynthetic capacity under heat stress, indicating the physiological basis for the heat tolerance of wheat imparted by delayed sowing.

Light energy captured by the photosynthetic apparatus is mainly used for photosynthetic electron transfer, but a small portion is dissipated in the form of fluorescence and heat energy; these three components compete and restrict one another (Li et al., 2005). The J-step and I-step of the OJIP standardized curve are related to the redox states of Q_*A*_ and plastoquinone, respectively (Strasser, 1997). In this study, the J-step and I-step in the fast chlorophyll fluorescence kinetic curve were higher under normal sowing than delayed sowing, indicating that the Q_*A*_–Q_*B*_ process of photosynthetic electron transfer in flag leaves was inhibited under heat stress with normal sowing. Previous studies also showed that heat stress influences electron transport, which damages the function of PS II (Chen et al., 2014; Bi et al., 2016; Fatma et al., 2021). And our research shows that RC/CSm, TRo/CSm, ETo/CSm, and DIo/CSm were lower under normal sowing than delayed sowing. The excess excitation energy of PSII increases, resulting in the production of a large number of reactive oxygen species (ROS), which damages the photosynthetic apparatus (Pavel, 2011; Takahashi and Badger, 2011). Our study shows that compared with the normal sowing, SOD, POD, CAT enzyme activity of winter wheat flag leaf to maintain at a high level, and reduce the MDA content under delayed sowing, that late sown with winter wheat flag leaf ROS removal ability is stronger, so as to keep the dynamic balance of its generation and removal, and reduce the level of membrane lipid peroxidation. Therefore, compared with normal sowing, delayed sowing ensured the energy transmission through enhancing the scavenging capacity of ROS in flag leaves, therefore increased the distribution of available energy in photochemistry and improved the assimilation capacity of photosynthetic system.

Studies have shown that, high temperature inhibits most proteins involved in chlorophyll synthesis, photosynthetic electron transport, and the Calvin cycle, thereby reducing photosynthesis in wheat leaves (Lu et al., 2017). Some researchers have speculated that heat adaptation before anthesis can effectively alleviate the photosynthetic and oxidative damage to wheat flag leaves from heat stress after anthesis (Wang et al., 2011), but a proteomics-based mechanism of action has not been reported. In our study, delayed sowing significantly upregulated the expression of GR protein under heat stress, while normal sowing significantly downregulated the expression of GR protein and D2 protein (PsbR). D1, D2 and oxygen-associated complex (OEC) proteins are sensitive sites in photosynthetic apparatus to environmental stress, most biological and abiotic stresses can inhibit their protein synthesis, resulting in protein degradation and ROS increase, leading to the intensification of photoinhibition (Besford et al., 1993; Takahashi and Murata, 2008; Shu et al., 2015). Therefore, we think that D1 and OEC proteins are also affected by ROS, leading to leaf senescence under normal sowing. Meanwhile, comparison of DEPs revealed that under heat stress, the sowing date affects many proteins related to photosynthesis. Compared with normal sowing, three proteins related to chlorophyll precursor synthesis, six proteins related to photosynthetic electron transfer, and three proteins related to the Calvin cycle were significantly increased in winter wheat leaves under delayed sowing. GO biological process and KEGG pathway enrichment analyses further revealed that proteins related to photosynthesis in winter wheat leaves increased significantly under delayed sowing. Therefore, delayed sowing upregulated the expression of psbR in D2 synthesis protein of wheat flag leaf, and protected D2 protein from heat stress by enhancing antioxidant capacity and stability of flag leaf membrane protein, thus delaying the senescence of wheat leaves and improving photosynthetic capacity under heat stress.

We mapped the DEPs in the metabolic pathway involved in photosynthesis in winter wheat leaves under delayed sowing using KEGG analysis. The protein contents of PsbH, PsbR, and PetB increased significantly. PsbR is an important part of the eukaryotic PSII, and is mainly involved in the oxidation reaction and electron transfer of PSII (Liu et al., 2009; Allahverdiyeva et al., 2013). PsbR stabilizes the PSII complex, affecting the properties of both the acceptor- and donor-side electron transfer reactions (Allahverdiyeva et al., 2007). Meanwhile, PsbH is a single transmembrane helix subunit necessary for the proper function of PSII and its stable assembly (D’Haene et al., 2015). A PsbH deletion mutant showed retardation of Q_*A*_–Q_*B*_ electron transfer (Mayes et al., 1993), and the absence of PsbH has also been shown to decrease the efficiency of the PSII repair cycle and cause oxidative damage to PSII proteins (Komenda et al., 2002; Bergantino et al., 2003). At present, studies have shown that Roles of Potassium silicate (K_2_SiO_3_, 1.5 mM) and silicon dioxide nanoparticles (SiO_2_NPs, 1.66 mM) in improving thermotolerance of wheat photosynthetic machinery via upregulation of PsbH, PsbB, and PsbD Genes encoding PSII core Proteins (Hassan et al., 2021). From a proteomics perspective, delayed sowing significantly increases the proteins related to photosynthetic electron transport (PsbH, PsbR, and PetB) under heat stress, thereby affecting the process of photosynthetic electron transport.

## Conclusion

Under heat stress during grain filling, delayed sowing date of winter wheat reduced heat dissipation by enhancing the scavenging capacity of ROS in flag leaves, and ensuring energy transmission along the photosynthetic electron transport chain; this increased the distribution ratio of available energy in photochemical reactions and maintained a high photosynthetic system assimilation capacity, which supported a high photosynthetic rate. Proteomic analysis revealed that differential accumulation of photosynthesis-related proteins, such as PsbH and PsbR, under delayed sowing in response to heat stress was coupled with physiological traits, confirming that delayed sowing can mediate tolerance to heat stress via improved photosynthetic electron transport to enhance the photosynthetic capacity. These findings provide a theoretical basis for the cultivation of heat tolerant winter wheat.

## Data Availability Statement

The datasets presented in this study can be found in online repositories. The names of the repository/repositories and accession number(s) can be found below: ProteomeXchange; PXD031871.

## Author Contributions

XD and MH designed the experiments and managed the projects. LF, JC, XZ, and SD performed the experiments. LF performed the data analysis and wrote the manuscript. All authors listed have approved the manuscript that is enclosed.

## Conflict of Interest

The authors declare that the research was conducted in the absence of any commercial or financial relationships that could be construed as a potential conflict of interest.

## Publisher’s Note

All claims expressed in this article are solely those of the authors and do not necessarily represent those of their affiliated organizations, or those of the publisher, the editors and the reviewers. Any product that may be evaluated in this article, or claim that may be made by its manufacturer, is not guaranteed or endorsed by the publisher.

## Footnotes

1
http://www.uniprot.org/

2
http://www.geneontology.org/

## Abbreviations

DEPs: differentially expressed proteins
OJIP: the chlorophyll a fluorescence transient
DAA: days after anthesis
RC/CSm: the amount of active PSII reaction centers per cross section
TRo/CSm: trapped energy flux per CSm
ETo/CSm: electron transport flux per CSm
DIo/CSm: dissipated energy flux per RC
Φ Po: the maximal photochemical efficiency of PSII
ψ o: excitation efficiency of electron transport beyond QA
Φ Eo: quantum yield for electron transfer
Φ Do: quantum ratio for heat dissipation
ROS: reactive oxygen species
SOD: superoxide dismutase
POD: peroxidase
CAT: catalase
MDA: malondialdehyde
GO: Gene Ontology
KEGG: Kyoto Encyclopedia of Genes and Genomes.
