# Supplementary material for: Physiological and Proteomic Analyses Indicate Delayed Sowing Improves Photosynthetic Capacity in Wheat Flag Leaves Under Heat Stress
Source: Front Plant Sci. 2022 Mar 24;13:848464. doi: 10.3389/fpls.2022.848464 (PMC8988879; doi:10.3389/fpls.2022.848464)
Supplement: Supplementary file 1 [file Data_Sheet_1.docx]

Supplementary Figure 1. Mean temperature (℃) and precipitation (mm) recorded during the growing seasons (from October 8 to June 9) in 2017–2018 and 2018–2019.

Humidity (%)

Humidity (%)

Humidity (%)

Humidity (%)

Time (h)

Supplementary Figure 2. The average humidity of the air at the flag leaf layer between delayed sowing and normal sowing during heat stress (19–21 DAA). A and B are delayed sowing and normal sowing in the 2017–2018 growing seasons respectively, C and D are delayed sowing and normal sowing in the 2018–2019 growing seasons respectively. (Note: NH, Natural humidity; HH, Humidity under heat stress)

Supplementary Figure 3. Wheat flag leaves temperature and air temperature during photosynthesis measurement at 20 DAA. (Note: Different letters denote statistical differences by LSD test (P < 0.05) between treatments for each parameter in the same year.)

Grain weight (mg)

Days after anthesis (d)

Supplementary Figure 4. Effects of delayed and normal sowing on grain weight during grain filling under heat stress in the 2018–2019 growing seasons. (Note: the red curve represents the fitting curve of delayed sowing grain weight, while the black curve represents the fitting curve of normal sowing grain weight.)


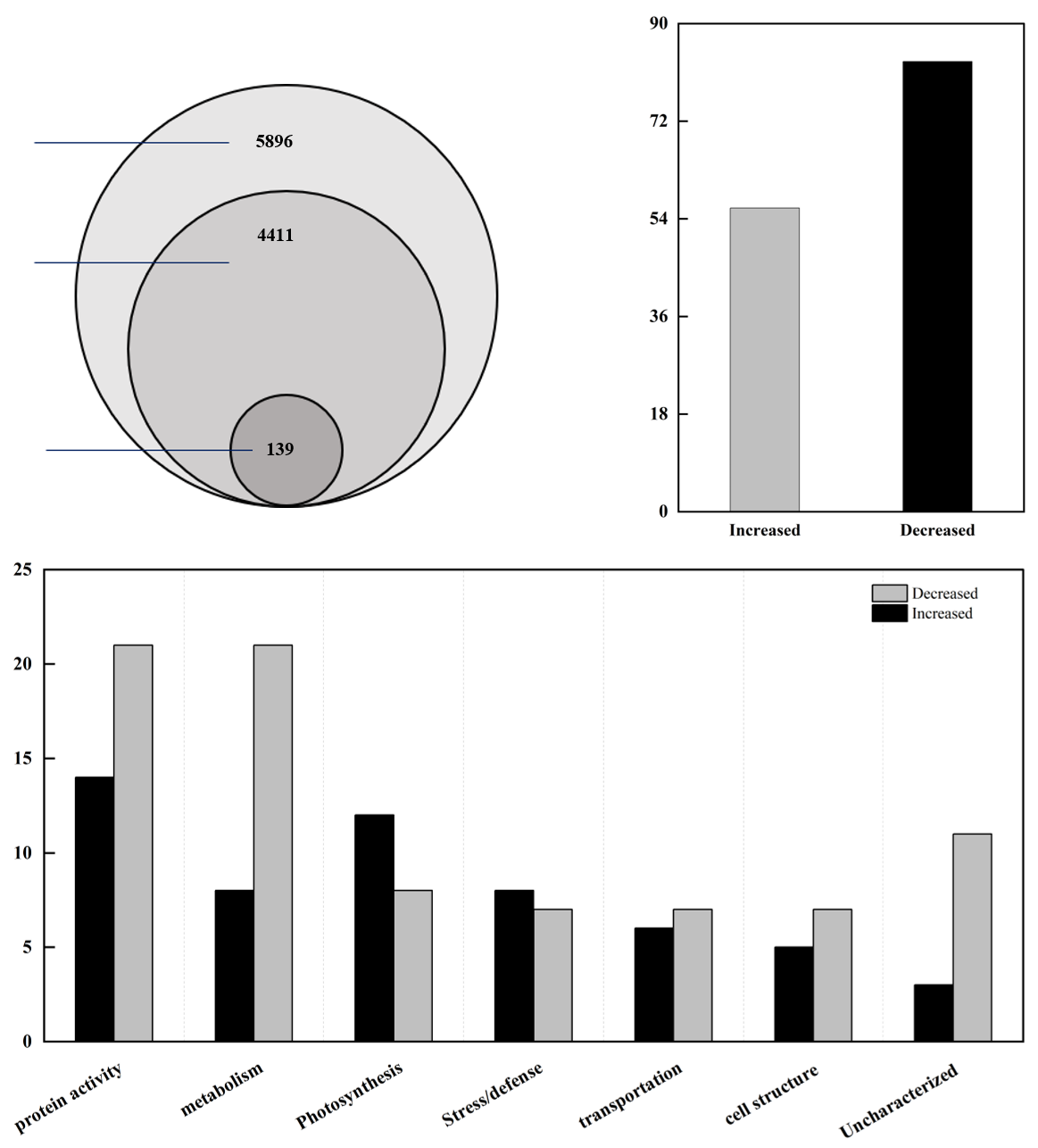


A

B

Identified

Quantitative

Differential

Protein number

C

Protein number

Supplementary Figure 5. Protein identification result statistics. (A) Schematic diagram of proteins characterization. (B) Number of differentially expressed proteins. (C) Functional classification of differentially expressed proteins.

Supplementary Table 1. The growth process of wheat under different treatments

| Year | Treatment | Sowing date | Seeding date | | Jointing date | Heading date | Anthesis | Mature date |
| --- | --- | --- | --- | --- | --- | --- | --- | --- |
| 2017–2018 | 8-Oct. | Oct. 8, 2017 | | Oct. 14, 2017 | Mar. 29, 2018 | Apr. 29, 2018 | May. 3, 2018 | Jun. 8, 2018 |
|  | 22-Oct. | Oct. 22, 2017 | | Oct. 29, 2017 | Mar. 31, 2018 | Apr. 31, 2018 | May. 4, 2018 | Jun. 8, 2018 |
| 2018–2019 | 8-Oct. | Oct. 8, 2018 | | Oct. 13, 2018 | Mar. 27, 2019 | Apr. 26, 2019 | May. 1, 2019 | Jun. 9, 2018 |
|  | 22-Oct. | Oct. 22, 2018 | | Oct. 28, 2018 | Mar. 29, 2019 | Apr. 27, 2019 | May. 2, 2018 | Jun. 9, 2018 |

Supplementary Table 2. Identification of differentially expressed proteins (DEPs) and Functional Classification. 139 proteins were assigned as DEPs (fold≥1.5, T-test p value < 0.05).

| **Protein accession** | **Protein description** | **22-Oct/8-Oct Ratio** | **22-Oct/8-Oct P value** |
| --- | --- | --- | --- |
| **cell structure** | | | |
| A0A3B6HTJ6 | Histone H2A | 4.55 | 0.000 |
| A0A3B6TWP1 | Histone H2A | 4.02 | 0.000 |
| A0A3B5Y3R2 | Uncharacterized protein | 1.82 | 0.002 |
| W5A761 | RPN13_C domain-containing protein | 1.56 | 0.039 |
| A0A3B5ZXN8 | Uncharacterized protein | 2.33 | 0.005 |
| Q43213 | Protein H2A.5 | 0.66 | 0.003 |
| S4Z0G4 | Ribosomal protein L32 | 0.65 | 0.007 |
| A0A3B6MZC2 | H15 domain-containing protein | 0.65 | 0.000 |
| A0A3B6DKU9 | H15 domain-containing protein | 0.61 | 0.002 |
| A0A3B6KR72 | H15 domain-containing protein | 0.57 | 0.001 |
| A0A3B6LV22 | H15 domain-containing protein | 0.52 | 0.006 |
| Q8LRU5 | HMG-I/Y protein HMGa | 0.48 | 0.000 |
| **protein synthesis/folding/degradation** | | | |
| A0A3B6ISA1 | RRM domain-containing protein | 2.93 | 0.001 |
| A0A3B6RSZ0 | AAA domain-containing protein | 2.84 | 0.000 |
| A0A3B6LMV5 | Protein kinase domain-containing protein | 2.12 | 0.014 |
| D4P3E6 | ARF GTPase activator (Fragment) | 1.96 | 0.013 |
| A0A3B5ZZN9 | Ribosomal_L28e domain-containing protein | 1.85 | 0.007 |
| A0A3B6GW29 | Uncharacterized protein | 1.81 | 0.032 |
| A0A3B6JF20 | Uncharacterized protein | 1.79 | 0.041 |
| A0A3B6GSR5 | Peptidase_M16 domain-containing protein | 1.76 | 0.007 |
| A0A3B6EPI8 | Uncharacterized protein | 1.72 | 0.036 |
| A0A3B6IVK1 | Uncharacterized protein | 1.68 | 0.033 |
| A0A3B6NUX8 | Uncharacterized protein | 1.62 | 0.011 |
| A0A3B5ZRI8 | Proteasome subunit beta | 1.60 | 0.006 |
| A0A3B6RL68 | Uncharacterized protein | 1.56 | 0.000 |
| E7D433 | EF-hand calcium-binding protein | 1.51 | 0.039 |
| A0A3B6A0C1 | Proline iminopeptidase | 0.67 | 0.015 |
| A0A3B6JH83 | Uncharacterized protein | 0.67 | 0.001 |
| A0A3B6I7N1 | Uncharacterized protein | 0.66 | 0.006 |
| A0A3B5YUX3 | Uncharacterized protein | 0.65 | 0.007 |
| A0A3B6HZL7 | Uncharacterized protein | 0.65 | 0.003 |
| A0A0C4BKI1 | Uncharacterized protein | 0.64 | 0.001 |
| A0A1D6BC85 | Ribosomal protein L37 | 0.63 | 0.005 |
| A0A3B6KPT6 | Uncharacterized protein | 0.63 | 0.043 |
| A0A3B6H3T7 | Amidohydro-rel domain-containing protein | 0.63 | 0.009 |
| A0A3B6SHC1 | AB hydrolase-1 domain-containing protein | 0.62 | 0.002 |
| A0A3B5XXY0 | Uncharacterized protein | 0.62 | 0.023 |
| A0A3B6KNG3 | Thioredoxin-like_fold domain-containing protein | 0.62 | 0.015 |
| A0A3B6LR88 | Uncharacterized protein | 0.60 | 0.038 |
| Q43665 | Wali5 protein | 0.59 | 0.006 |
| A0A3B5XY55 | Uncharacterized protein | 0.59 | 0.040 |
| A0A3B6TIW7 | ABC1 domain-containing protein | 0.57 | 0.000 |
| Q43202 | Histone H3 (Fragment) | 0.57 | 0.000 |
| W5H631 | 60S ribosomal protein L27 | 0.56 | 0.000 |
| A0A3B6EK52 | Uncharacterized protein | 0.54 | 0.028 |
| A0A3B6HRQ6 | SAP domain-containing protein | 0.52 | 0.000 |
| A0A3B6BZC4 | Uncharacterized protein | 0.50 | 0.002 |
| **Metabolism** | | | |
| A0A077S0D9 | Str_synth domain-containing protein | 2.78 | 0.004 |
| A0A3B6A1R3 | Uncharacterized protein | 2.44 | 0.013 |
| A0A3B6BZV3 | Uncharacterized protein | 1.66 | 0.013 |
| A0A3B6GW22 | GP-PDE domain-containing protein | 1.64 | 0.042 |
| A0A3B6LEZ6 | Uncharacterized protein | 1.60 | 0.005 |
| S4Z3H9 | "30S ribosomal protein S11, chloroplastic" | 1.60 | 0.041 |
| A0A3B6KRR5 | Uncharacterized protein | 1.53 | 0.006 |
| A0A3B6GS53 | Aconitate hydratase | 1.51 | 0.008 |
| W5D2I6 | Non-specific lipid-transfer protein | 0.66 | 0.021 |
| A0A3B6U9K3 | Glycosyltransferase | 0.66 | 0.028 |
| Q5I7K5 | Ribosomal protein P1 | 0.66 | 0.021 |
| A0A3B6MLK6 | Pyruvate dehydrogenase E1 component subunit beta | 0.65 | 0.025 |
| Q9ARG8 | Sucrose-6F-phosphate phosphohydrolase SPP3 | 0.65 | 0.037 |
| A0A3B5YPS5 | 4-hydroxyphenylpyruvate dioxygenase | 0.64 | 0.001 |
| A0A3B5ZY48 | Amidophosphoribosyltransferase | 0.64 | 0.004 |
| A0A3B6EEC9 | AB hydrolase-1 domain-containing protein | 0.64 | 0.042 |
| A0A077RTX0 | Glycosyltransferase | 0.64 | 0.048 |
| A0A3B5YZL3 | Uncharacterized protein | 0.63 | 0.000 |
| A0A3B6PK79 | Fumarylacetoacetase | 0.63 | 0.005 |
| A0A3B6MST1 | Peptidylprolyl isomerase | 0.63 | 0.001 |
| A0A3B6QIQ1 | Cellulase domain-containing protein | 0.62 | 0.003 |
| A0A3B6QIE7 | Uncharacterized protein | 0.61 | 0.005 |
| A0A3B6SNM1 | Uncharacterized protein | 0.61 | 0.000 |
| A0A3B6LSQ4 | Ornithine aminotransferase | 0.61 | 0.009 |
| A0A3B6EI74 | Malic enzyme | 0.57 | 0.000 |
| W5HTZ1 | Uncharacterized protein | 0.57 | 0.007 |
| A0A3B6H4C8 | Uncharacterized protein | 0.52 | 0.000 |
| A0A3B5XW39 | CYTOSOL_AP domain-containing protein | 0.48 | 0.017 |
| A0A3B6KKA8 | AP2/ERF domain-containing protein | 0.44 | 0.009 |
| **Photosynthesis/Respiration** | | | |
| S4Z3A1 | Photosystem II reaction center protein H | 2.08 | 0.000 |
| A0A3B6MXQ2 | FMN hydroxy acid dehydrogenase domain-containing protein | 2.04 | 0.014 |
| A0A3B6NU25 | Uncharacterized protein | 1.92 | 0.001 |
| A0A3B6QKC9 | PKS_ER domain-containing protein | 1.71 | 0.020 |
| A0A3B6EN87 | CAAD domain-containing protein | 1.63 | 0.013 |
| A0A3B6JJ08 | ATPase_AAA_core domain-containing protein | 1.57 | 0.000 |
| A0A3B6LW30 | Mg-protoporphyrin IX chelatase | 1.56 | 0.003 |
| A0A2P0ZG21 | Ribulose bisphosphate carboxylase large chain (Fragment) | 1.53 | 0.004 |
| A0A3B6NW49 | Uncharacterized protein | 1.53 | 0.010 |
| A0A3B6U5D2 | "Chlorophyll a-b binding protein, chloroplastic" | 1.52 | 0.002 |
| A0A3B5Z1L6 | Uncharacterized protein | 1.51 | 0.028 |
| P60162 | Cytochrome b6 | 1.51 | 0.000 |
| A0A3B6MQD6 | Uncharacterized protein | 0.63 | 0.043 |
| A0A3B6LKD1 | PAP_fibrillin domain-containing protein | 0.57 | 0.014 |
| A0A218LW52 | NADH-plastoquinone oxidoreductase subunit I (Fragment) | 0.56 | 0.000 |
| A0A3B5XW56 | Uncharacterized protein | 0.54 | 0.002 |
| A0A3B6C222 | PAP_fibrillin domain-containing protein | 0.46 | 0.000 |
| A0A1D5UUP8 | PAP_fibrillin domain-containing protein | 0.44 | 0.003 |
| A0A3B6RNY5 | Uncharacterized protein | 0.28 | 0.000 |
| A0A3B6TNU5 | Uncharacterized protein | 0.21 | 0.018 |
| **Stress/defense/detoxification** | | | |
| C4PW04 | MPBQ methyltransferase (Fragment) | 2.85 | 0.005 |
| A0A1D5UPW1 | FAD-binding FR-type domain-containing protein | 2.70 | 0.020 |
| A0A3B6JM67 | Glutathione reductase | 1.83 | 0.025 |
| A0A3B6C0V7 | Uncharacterized protein | 1.82 | 0.010 |
| A0A3B6H702 | Uncharacterized protein | 1.80 | 0.023 |
| A0A3B6SF84 | Uncharacterized protein | 1.66 | 0.034 |
| A0A3B6PNM7 | Annexin | 1.60 | 0.016 |
| A0A3B6MI27 | Uncharacterized protein | 1.59 | 0.005 |
| C6ETB3 | Peroxidase | 0.66 | 0.009 |
| Q75QN8 | Cold shock domain protein 3 | 0.64 | 0.015 |
| S6AWC2 | Cold induced 16 | 0.63 | 0.001 |
| A0A3B6D6R2 | Peroxidase | 0.59 | 0.001 |
| A0A3B6KST8 | Uncharacterized protein | 0.57 | 0.000 |
| B5A8A6 | Glutaredoxin | 0.52 | 0.023 |
| A0A3B6JLW9 | Uncharacterized protein | 0.50 | 0.002 |
| A0A3B6RMX5 | Uncharacterized protein | 0.40 | 0.000 |
| **transportation** | | | |
| A0A3B6A2U5 | GTP-binding nuclear protein | 2.80 | 0.002 |
| A0A3B6RN65 | Type I inorganic proton-pumping pyrophosphatase 2-A | 2.78 | 0.027 |
| A0A3B6QJF9 | Uncharacterized protein | 2.09 | 0.001 |
| A0A3B6SP42 | Type I inorganic proton-pumping pyrophosphatase 2-B | 1.72 | 0.011 |
| A0A3B6JR02 | Uncharacterized protein | 1.70 | 0.007 |
| A0A3B6MRB1 | Uncharacterized protein | 1.65 | 0.001 |
| A0A3B6DA83 | MFS domain-containing protein | 0.66 | 0.047 |
| A0A3B6QLZ6 | Uncharacterized protein | 0.64 | 0.000 |
| A0A3B5ZTI0 | DUF1338 domain-containing protein | 0.63 | 0.012 |
| W5FLI7 | V-type proton ATPase proteolipid subunit | 0.61 | 0.023 |
| A0A3B6JRM6 | Uncharacterized protein | 0.60 | 0.008 |
| Q41629 | "ADP,ATP carrier protein 1, mitochondrial" | 0.59 | 0.011 |
| A0A3B6NUV0 | Uncharacterized protein | 0.54 | 0.027 |
|  |  |  |  |
| **Uncharacterized protein** | | | |
| A0A3B6GRZ7 | Uncharacterized protein | 3.03 | 0.000 |
| A0A3B6KTA7 | Uncharacterized protein | 2.07 | 0.039 |
| A0A3B6JIS6 | Uncharacterized protein | 1.70 | 0.017 |
| A0A3B6TS71 | Uncharacterized protein | 0.66 | 0.032 |
| A0A3B6QJF6 | Uncharacterized protein | 0.65 | 0.000 |
| A0A3B6RLF3 | UBIQUITIN_CONJUGAT_2 domain-containing protein | 0.65 | 0.001 |
| A0A3B6ELW1 | DUF1794 domain-containing protein | 0.64 | 0.019 |
| A0A3B6JEL3 | Uncharacterized protein | 0.63 | 0.005 |
| A0A3B6U498 | Uncharacterized protein | 0.61 | 0.001 |
| A0A3B6H6S6 | Uncharacterized protein | 0.60 | 0.003 |
| A0A3B5YWB4 | DUF1338 domain-containing protein | 0.59 | 0.008 |
| W5CPY8 | Uncharacterized protein | 0.54 | 0.034 |
| A0A3B6B301 | Uncharacterized protein | 0.52 | 0.008 |
| A0A3B6EBI2 | Peptidase A1 domain-containing protein | 0.52 | 0.001 |
